# Continuous Detection of Increasing Concentrations of Thrombin Employing a Label-Free Photonic Crystal Aptasensor

**DOI:** 10.3390/mi11050464

**Published:** 2020-04-28

**Authors:** Paula Martínez-Pérez, Maribel Gómez-Gómez, Todora Angelova, Amadeu Griol, Juan Hurtado, Laurent Bellieres, Jaime García-Rupérez

**Affiliations:** Nanophotonics Technology Center, Universitat Politècnica de València, Camino de Vera s/n, 46022 Valencia, Spain; paumarp3@ntc.upv.es (P.M.-P.); migmegme@ntc.upv.es (M.G.-G.); tivanova@ntc.upv.es (T.A.); agriol@ntc.upv.es (A.G.); juahurmo@ntc.upv.es (J.H.); blaurent@ntc.upv.es (L.B.)

**Keywords:** photonic crystal, photonic bandgap, optical biosensor, aptasensor, label-free, thrombin

## Abstract

Thrombin generation is a complex and finely regulated pathway that provokes dynamical changes of thrombin concentration in blood when a vascular injury occurs. In order to characterize the initiation phase of such process, when thrombin concentration is in the nM range, a label-free optical aptasensor is proposed here. This aptasensor combines a 1D photonic crystal structure consisting of a silicon corrugated waveguide with thrombin binding aptamers on its surface as bioreceptors. As a result, this aptasensor has been demonstrated to specifically detect thrombin concentrations ranging from 270 pM to 27 nM with an estimated detection limit of 33.5 pM and a response time of ~2 min. Furthermore, it has also been demonstrated that this aptasensor is able to continuously respond to consecutive increasing concentrations of thrombin and to detect binding events as they occur. All these features make this aptasensor a good candidate to continuously study how thrombin concentration progressively increases during the initiation phase of the coagulation cascade.

## 1. Introduction

Thrombin is a pleiotropic enzyme with a central role in blood coagulation. In normal conditions it is absent in blood. However, when a vascular injury occurs, the coagulation cascade is activated and thrombin is generated from its precursor plasma protein, prothrombin, to convert fibrinogen to fibrin, which aggregates together with platelets creating clots that avoid blood losses [1]. Thrombin generation is divided into three consecutive stages characterized by the factors involved and the final thrombin concentration achieved: initiation (from 1 nM to 20–30 nM thrombin), propagation (from 20–30 nM up to more than 800 nM thrombin), and termination (when thrombin is no longer produced and free thrombin is inactivated) [2,3,4,5,6]. Such a complex process is known as vascular hemostasis and the disruption of this hemostatic state is related to the onset of diseases such as thrombosis; atherosclerosis [7]; Alzheimer’s [8]; or growth, metastasis, and angiogenesis of tumors [9,10].

Consequently, in recent years, a great interest has arisen in order to develop systems able to monitor variations in thrombin concentration accurately. Currently, some thrombin generation assays are commercially available. However, most of them are based on indirect measurements and the use of labeled substrates. Additionally, they require laborious preparation, cannot assess all the phases of hemostasis, and the lack of standardization prevents their use in general clinical practice [11,12,13]. To overcome such limitations, aptamer binding assays have come to the attention of researchers as a suitable approach due to the outstanding features of aptamers as biorecognition molecules [14]. Aptamers are single-stranded DNA or RNA molecules that can bind their targets with high affinity and specificity. This is due to the in vitro process used for their chemical synthesis, known as Systematic Evolution of Ligands by Exponential Enrichment (SELEX), which allows producing those aptamers at large scales with very little activity variation among batches. In addition to their binding capabilities, aptamers are also more stable in extreme media than typically used antibodies as they exhibit reversible folding and can be easily chemically modified [15,16].

These promising features of aptamers explain their employment in the development of aptasensors: biosensors whose biorecognition element is an aptamer. They are used to selectively and specifically detect the presence of tiny amounts of analytes of interest in applications related to medical diagnosis [17], environmental monitoring [18], or biodefense [19,20], among others. There are several kinds of aptamers and transducers, whose combination results in a huge variety of aptasensors that are usually classified according to the transducer working principle. Among all of them, optical transducers exhibit outstanding characteristics such as high sensitivity, multiplexing capability, direct and label-free real-time detection, miniaturization possibilities, immunity to electromagnetic interferences, and cost-effectiveness [21]. 

Considering the outstanding features of aptamers and optical transducers, the aim of this work is developing an optical aptasensor based on photonic crystal (PC)-sensing structures for the detection of increasing concentrations of thrombin. The selected PC-sensing structures consist of a silicon 1D periodic corrugated waveguide [22], whose photonic band gap (PBG) in the transmission spectrum will shift when the target thrombin molecules are specifically recognized by the aptamers immobilized on its surface [23]. Regarding those aptamers, a well-known thrombin binding aptamer (TBA) consisting of a single-stranded DNA with a 15-mer sequence and a high affinity towards thrombin was chosen [24,25]. Regarding the application, we will focus on studying the initiation phase of the thrombin generation process. At this stage, very reduced thrombin concentrations (ranging from 1 nM to 20–30 nM) are produced to trigger the conversion of fibrinogen to fibrin and hence, the clotting process [4,5,6]. However, typically used clinical tests to study thrombin generation are not sensitive enough to detect this thrombin concentration range [3]. Some works have reported the development of aptasensors able to detect lower concentrations of thrombin, for example, in research of Chen, X. et al. [26] and Cho, H. et al. [27]. However, they require the incubation of thrombin and the TBA as well as the use of dyes to measure the final TBA-thrombin complexes, thus not offering the possibility of performing a continuous and label-free monitoring of the binding events as reported in the current work.

Combining both TBA and our PC structures, it is demonstrated that a direct detection of thrombin concentrations as low as 270 pM can be performed by monitoring in real time the binding events while they occur. This aptasensor has a sensitivity of 12.6 pm/nM in its linear range (below 2.7 nM thrombin), an estimated limit of detection (LOD) of 33.5 pM, and an average response time of ~2 min. Furthermore, it is also evinced that a continuous increase of the concentration of thrombin can be continuously detected, which makes this optical aptasensor a good candidate to monitor the formation of thrombin during the initiation phase of the thrombin generation process.

## 2. Materials and Methods 

### 2.1. Materials

3-Aminopropyltriethoxysilane (APTES), α-human thrombin, lyophilized bovine serum albumin (BSA) protein, Phosphate-Buffered Saline (PBS) 10× solution, and 1 M MgCl_2_ solution were purchased from Fisher Scientific (Waltham, MA, USA). Twenty-five percent glutaraldehyde solution was purchased from Sigma-Aldrich (San Luis, MO, USA). A 15-mer thrombin binding aptamer (TBA) with a sequence of 5’–NH2–(T)_12_–GGTTGGTGTGGTTGG–3’ was purchased from NZYTech, Lda (Lisbon, Portugal). Absolute ethanol, acetone, 47% hydrofluoric acid (HF) solution, hydrogen peroxide, sulfuric acid, and isopropyl alcohol (IPA) were purchased from VWR (Radnor, PA, USA). 

### 2.2. Fabrication of PC Structures

**Figure 1:**
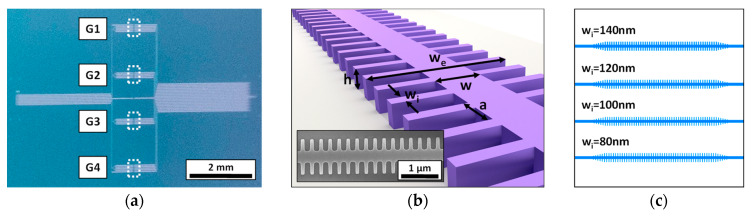
(**a**) Image of the fabricated photonic chip, where the different groups of 1D photonic crystal (PC) sensing structures are indicated. (**b**) Sketch of the 1D PC structure showing its main parameters. The inset shows a Scanning Electron Microscopy (SEM) image of one of the fabricated 1D PC sensing structures. (**c**) Distribution of the 1D PC structures within each sensors group.

The employed 1D PC corrugated waveguides have been fabricated on a silicon-on-insulator (SOI) wafer employing e-beam lithography (EBL) and inductively coupled plasma etching of the top silicon layer (see Figure 1a). The parameters of the 1D PC structures are the following (see Figure 1b); height (h) of 220 nm, width (w) of 460 nm, period (a) of 380 nm, transversal elements length (w_e_) of 1500 nm, and four possible transversal elements widths (w_i_): 140, 120, 100, or 80 nm. In order to improve light coupling to/from the 1D PC structures and the input/output single mode waveguides (also with a width of 460 nm), a 5-element taper was included. These silicon integrated 1D PC structures are highly compact (footprint below 50 µm^2^), thus offering the opportunity to create arrays of sensors within the same photonic chip. Accordingly, in this work, we have designed a photonic chip containing 16 PC sensing structures distributed in 4 groups (named G1, G2, G3, and G4) of 4 PC sensors with the different w_i_ parameters previously indicated (see Figure 1c). Besides the PC sensors groups, alignment reference waveguides were also included in the chip. All the photonic structures within the chip were accessed at the input and the output via 70-nm-deep shallow etched 1D grating couplers.

### 2.3. Biofunctionalization of PC Structures with Aptamers

In order to clean and activate the silicon surface of the PC structures, the chip is immersed in piranha solution (H_2_SO_4_:H_2_O_2_, 3:1) at 100 °C for 30 min, rinsed with deionized water, and dried with nitrogen. Afterwards, to perform the surface silanization, the chip is immersed in 1% APTES solution in 95% ethanol aqueous solution at 70 °C for 1 h. Then, it is rinsed three times with 95% ethanol and IPA, rinsed with water, dried with nitrogen, and cured for 15 min at 110 °C. Once silanized, the chip is immersed in a 2.5% glutaraldehyde solution in 1× PBS for 1 h at room temperature and then rinsed three times with 1× PBS and dried with compressed air. Finally, 5 µM TBA in folding buffer (1× PBS, 1 mM MgCl_2_) is dropped on the PC structures and allowed to react for 4 h at room temperature in humid atmosphere. Then, the chip is placed at 4 °C overnight. After incubation, the chip is rinsed with 1× PBS three times to remove the excess of TBA, dried with compressed air, and stored at 4 °C until use. Aptamers are pre-folded before incubation on the surface by heating the 5 µM TBA solution in folding buffer at 95 °C for 5 min and then allowing it to cool down at room temperature.

### 2.4. Surface Regeneration of PC Structures

In order to remove the biological layer created on the surface of the PC sensing structures after the performance of the biosensing experiments and being able to reuse them, the chip is regenerated by immersing it in piranha solution (H_2_SO_4_:H_2_O_2_, 3:1) for 30 min, rinsing it with deionized water, and drying with nitrogen. Then, it is rinsed with acetone and IPA, dried with nitrogen, and exposed to O_2_ plasma for 10 min. Finally, the chip is exposed to vapor of 16% HF aqueous solution for 7 s, rinsed with water, dried with nitrogen, and stored in desiccator until use.

### 2.5. Microfluidic Chamber

A microfluidic chamber is assembled on the chip in order to perform real-time biosensing experiments. Such microfluidic chamber consists of a PMMA piece and a double-sided adhesive tape with an open area on it (250 µm height, 8 mm length and 500 µm width). To assemble the fluidic system, the adhesive tape is glued on top of the silicon chip so that its open area covers the PC structures. Then, the PMMA piece with inlet and outlet tubing is properly attached to the adhesive tape in order to create a channel to flow liquids over the PC sensors. For pumping, a syringe pump working in withdraw mode is connected to the outlet tube and set at a constant rate of 20 µL/min.

### 2.6. Optical Setup for Biosensing Measurements

The optical set-up previously described in research of Ruiz-Tórtola, A. et al. [28] is used to perform the experiments. Briefly, it consists of a wavelength-tunable laser (81980A, Keysight, Santa Rosa, CA, USA), which is coupled to the input grating couplers of the chip through a fiber aspheric collimator (CFS2-1550-APC, Thorlabs, Newton, NJ, USA). The output light from the chip is collected by a 20× objective (Plan Achromat 0.4 N.A., Olympus, Shinjuku, Japan) connected to an infrared (IR) camera (Xeva-1.7-320, Xenics, Heverlee, Belgium). All these devices are controlled using a software implemented in LabVIEW (2014 SP1, National Instruments, Austin, TX, USA). 

### 2.7. Signal Processing and Data Analysis

In order to analyze the data obtained in the experiments, Matlab (R2019b, MathWorks, Natick, MA, USA) is used. First, fast Fourier transform (FFT) is applied to raw data. Then, inverse FFT is performed to data whose frequencies are located under a normalized cutoff frequency of 0.02. Once the original data is filtered, the local maximum transmission peak at the end of the PBG is located and fitted with a Gaussian function in order to determine its center position with a higher accuracy. This procedure is carried out automatically for all the spectra acquired during the experiments in order to determine the shift of the PBG edge.

## 3. Results and Discussion

### 3.1. Real-Time Detection of 27 nM and 2.7 nM Thrombin Solutions

**Figure 2:**
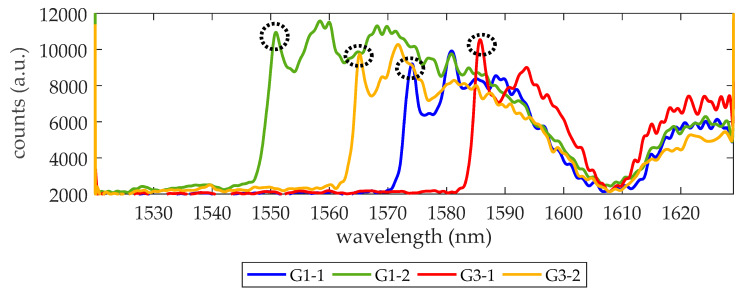
Transmission spectra of the four PC structures being monitored. The circles indicate the photonic band gap (PBG) edge, where a local maximum transmission is observed. The wavelength position of such maximum is tracked to know how the PBG edge shifts during biosensing experiments. Counts refer to the digital values provided by the 14-bit analog to digital converter (ADC) of the infrared (IR) camera. The dip around 1608 nm indicates the minimum for the response of the access grating couplers.

We begin trying to demonstrate the detection of a thrombin concentration of 27 nM (1 µg/mL), a value that is near the upper concentration of the initiation phase [4]. To this aim, the surface of the PC sensing structures in the chip is biofunctionalized as described in Section 2.3. Then, the microfluidic cell is attached to the photonic chip and the optical response of each sensing structure is checked while flowing 1× PBS. In this chip (hereafter referred as experimental chip), only PC sensors groups G1 and G3 have an acceptable transmission spectrum and are considered in the experiment. From these two PC groups, only the sensing response of the PC structures with w_i_ values 140 nm and 120 nm are monitored as their PBG edges are in the working wavelength range of the laser (from 1520 to 1630 nm) and have a well-defined edge shape (see Figure 2). Hereafter, the monitored PBG edges from PCs group G1 will be referred as G1-1 (w_i_ = 140 nm) and G1-2 (w_i_ = 120 nm) and those from PCs group G3 will be referred as G3-1 (w_i_ = 140 nm) and G3-2 (w_i_ = 120 nm). While flowing 1× PBS, the noise of the set-up was also characterized and a value of 8.9 pm was obtained. 

**Figure 3:**
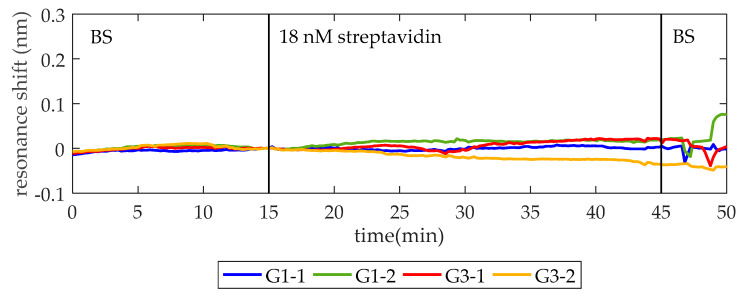
To test the recognition specificity, 18 nM streptavidin in blocking solution (BS) is flowed over the thrombin binding aptamer (TBA) functionalized photonic chip. In minute 15, after establishing a baseline with BS, the streptavidin solution is injected. One minute later (the time required for the solutions to reach the sensor form the syringe pump), it entered into the fluidic chamber and no change related to detection is observed, confirming that the surface is totally blocked and no unspecific recognition occurred.

Prior to thrombin detection, a blocking solution (BS) of 15 µM BSA in 1× PBS is flowed for 30 min to block the remaining reactive sites of glutaraldehyde on the surface (hereafter this process will be referred to as blocking). Afterwards, in order to check that the surface is completely blocked and no unspecific recognition by TBA occurs, a solution of 18 nM (1 µg/mL) streptavidin in BS is flowed. As it can be seen in Figure 3, when the streptavidin solution is flowed over the photonic chip, no changes occur in the position of the PBG edges, thus confirming that streptavidin is neither binding to the aptamers nor adsorbing to the surface.

**Figure 4:**
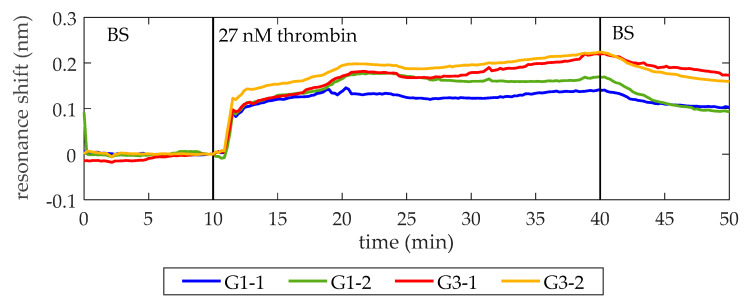
Sensing response of the PC sensing structures when a 27 nM thrombin solution is flowed. At minute 10, 27 nM thrombin solution in BS is flowed (it reaches the photonic chip one minute later) and its binding to TBA on the surface is clearly observed. At minute 40, BS is flowed again to remove the excess of thrombin. An average spectral shift of 97 pm and 166 pm is observed for the structures from groups G1 and G3, respectively. The bumps that appear while flowing 27 nM thrombin are ascribed to fluidic irregularities.

Once the lack of unspecific recognition is demonstrated, the BS is flowed again. When a stable baseline (i.e., centered in zero for at least 2 min and with a standard deviation below setup noise) is achieved, a solution of 27 nM thrombin in BS is flowed over the PC structures. As shown in Figure 4, a shift of PBG edges towards longer wavelengths is produced, indicating the binding of thrombin to the aptamers on the surface. Finally, BS is flowed again to remove the excess of thrombin. The average net shift of G1-1 and G1-2 is 97 pm, whereas the average net shift of G3-1 and G3-2 is 166 pm. The differences between PCs groups can be attributed to two factors. First, a different density of bioreceptors on their surface [29,30]. The biofunctionalization process is carried out manually, which might imply errors related to the manipulation of the materials employed, humidity, and temperature changes of the room and the precision when dropping the TBA solution over the PC structures. Second, slight structural differences between PCs groups are also expected due to the inherent limitations of EBL technique when fabricating the smallest features of the photonic chip [31]. These structural differences might be influencing the optical response of the PC structures and their sensitivity.

After this experiment, a negative control assay is performed to check unspecific adsorption of thrombin on the sensor surface. In parallel to the biofunctionalization of the experimental chip, another chip with the same characteristics (hereafter referred as control chip) is biofunctionalized using the same protocol, except that only PCs groups G1 and G2 are incubated with TBA while PCs groups G3 and G4 are incubated just with folding buffer (control groups). As in the previous experiment, the microfluidic flow cell is assembled on the photonic chip and the PBG edges monitored are G1-1, G1-2, G3-1, and G3-2. 

**Figure 5:**
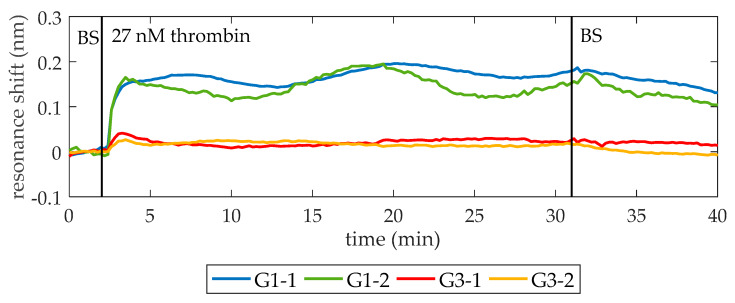
Sensing response obtained when flowing a 27 nM thrombin solution over a photonic chip where only half of their PC structures have TBA on their surface (G1-1 and G1-2). Only the PBG edges of such PC structures experience an average shift of 119 pm, whereas control PC structures (G3-1 and G3-2, without TBA) do not experience any sensing change. This confirms the recognition specificity. Regarding the instability of G1-1 and G1-2 while thrombin is flowed, it is again attributed to fluidic irregularities.

As in the previous experiment, PCs surfaces are blocked by flowing BS first. Once a stable baseline is obtained for BS, 27 nM thrombin in BS is introduced. As it can be observed in Figure 5, only those PC structures with the TBA immobilized on their surface exhibit a change in their PBG position when thrombin is flowed (an average net shift of 119 pm is observed), while no response is observed for those PC structures where the bioreceptors are not present, thus confirming that unspecific adsorption is not produced. Regarding the small peak observed when thrombin gets to the control PC structures, it indicates a RI increase attributed to flowing irregularities provoked by the contrast of densities of the solutions being exchanged in the fluidic chamber; once the new solution replaces the previous one and the flow regime is recovered, the PBG edges position is also recovered [23,32]. 

**Figure 6:**
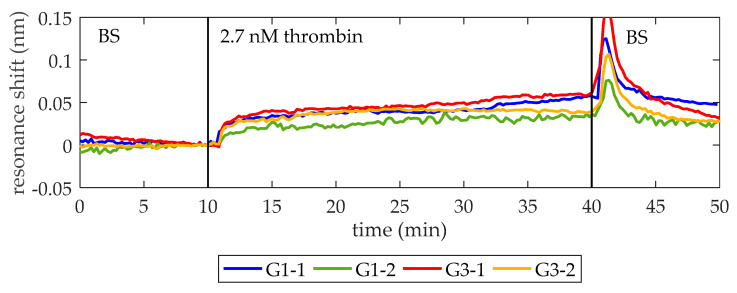
Sensing response of the PC-sensing structures when a 2.7 nM thrombin solution is flowed. At minute 10, 2.7 nM thrombin solution in BS is flowed (it reaches the photonic chip one minute later) and its binding to TBA on the surface is clearly observed. At minute 40, BS is flowed again to remove the excess of thrombin. The sudden peak observed is a RI increase due to different densities of the two solutions being exchanged in the fluidic chamber, as previously observed in Figure 5. An average spectral shift of 39 pm and 30 pm is observed for the structures from groups G1 and G3, respectively. Note that now the structure G1-2 corresponds to the 1D PC with w_i_ = 100 nm from group G1.

Once it was demonstrated that the optical aptasensor is able to specifically detect a thrombin concentration as low as 27 nM, the next objective is to detect a thrombin concentration near the lower concentration limit of the initiation phase (i.e., ~1 nM). With this aim, the experimental chip is regenerated using the protocol explained in Section 2.4 and biofunctionalized again as exposed in Section 2.3. Figure 6 shows the sensing results obtained when a 2.7 nM thrombin solution in BS is flowed over the photonic sensing chip (a ten-fold lower concentration with respect to previous experiments). Note that the thrombin is clearly detected despite its low concentration, providing average net shifts of 39 pm and 30 pm for PCs groups G1 and G3, respectively. In this occasion, differences between PCs groups are mainly due to the slightly higher desorption observed for G3-1 when BS is flowed again. As the photonic chip and the microfluidic chamber are the same employed for the detection of 27 nM thrombin solution, this different behavior can be ascribed to heterogeneities in the manually performed biofunctionalization process, as previously explained. 

### 3.2. Continuous Detection of Thrombin Level Increase

**Figure 7:**
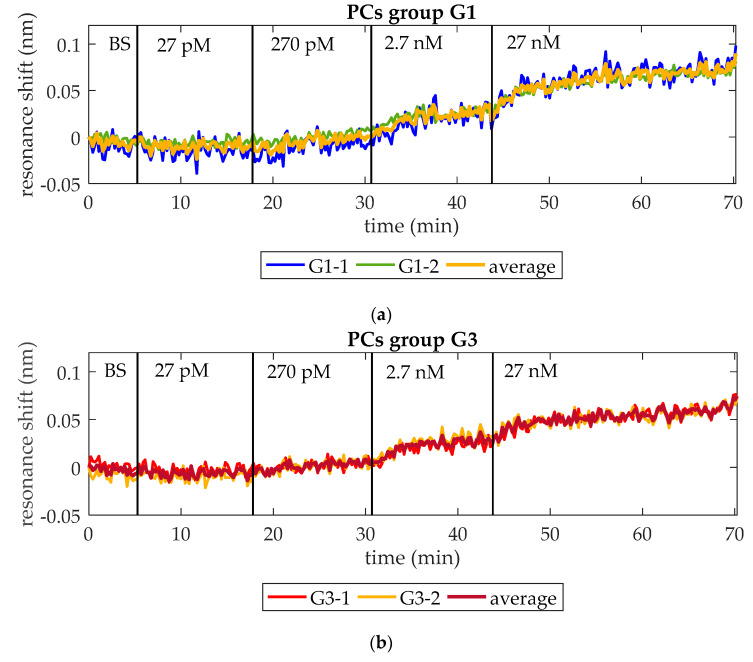

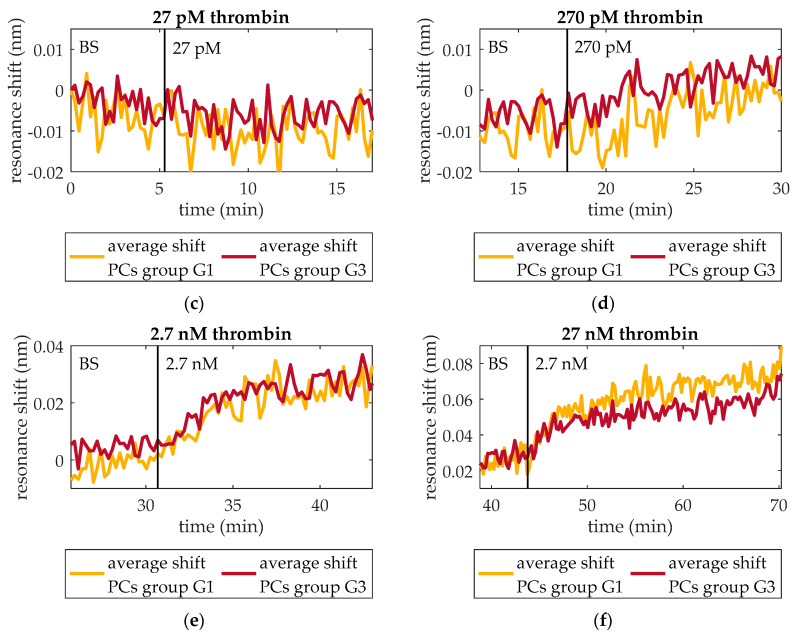
Resonance shift experienced by PCs groups G1 and G3 when increasing concentrations of thrombin are consecutively flowed over time. (**a**) Shift experienced by PCs group G1 and their average shift. (**b**) Shift experienced by PCs group G3 and their average shift. (**c**) Thrombin (27 pM) solution is not detected neither by PCs group G1 nor by group G3. (**d**) Thrombin (270 pM) solution provokes an average PBG shift of 12 pm for both PCs groups. (**e**) Thrombin (2.7 nM) solution provokes an average PBG shift of 24 pm and 22 pm for PCs groups G1 and G3, respectively. (**f**) Thrombin (27 nM) solution provokes an average PBG shift of 52 pm and 44 pm for PCs groups G1 and G3, respectively.

At this point, the capability of the optical aptasensor to specifically detect thrombin at concentrations as low as those observed during the initiation phase of the thrombin generation process has been demonstrated. The goal of this assay is assessing its capability to do this in a continuous way when the concentration is constantly increasing. With this aim, ten-fold serial dilutions of an initial 27 nM thrombin solution in BS are flowed over a photonic chip functionalized as explained in Section 2.3. As in previous assays, the PBG edges monitored are from PCs group G1 (G1-1 and G1-2) and PCs group G3 (G3-1 and G3-2). Figure 7 shows the experimental results obtained when increasing concentrations of thrombin are flowed over the photonic sensing chip. In Figure 7a, the evolution of the wavelength position of G1-1 and G1-2 is shown. An average shift of 12 pm, 24 pm, and 52 pm is observed when flowing 270 pM, 2.7 nM, and 27 nM thrombin solutions, respectively (see detail in Figure 7c–f). No change in the position of those PBG edges is observed when 27 pM thrombin solution is flowed. Regarding G3-1 and G3-2, they experienced an average shift of 12 pm, 22 pm, and 44 pm in presence of 270 pM, 2.7 nM, and 27 nM thrombin solutions, respectively (see detail in Figure 7c–f). These PBG edges did not change their position when flowing 27 pM thrombin solution either. It is important to remark that the response of both PCs groups is very similar in this assay, which indicates a good homogeneity regarding the optical behavior, the bioreceptor density on sensing structures and the transport of analyte from bulk solution to the sensors surface. Additionally, it is also demonstrated that a concentration as low as 270 pM can be detected. Therefore, this aptasensor shows a good response to increasing thrombin concentrations in a range that would allow to monitor the initiation phase of the thrombin generation process in real-time.

It is also remarkable that these results are consistent to those obtained from detection assays of discrete concentrations in Section 3.1. In this assay, when flowing 2.7 nM thrombin, the average total net shift showed by PCs group G1 is 36 pm and by PCs group G3 is 34 pm, similar to those observed in Figure 6 (39 pm and 30 pm, respectively). When flowing 27 nM, PCs group G1 shows an average total net shift of 88 pm, which is also very similar to that observed in Figure 4 (97 pm). Only PCs group G3 shows an average total net shift notably different from that observed in Figure 4 (78 pm vs. 166 pm). This difference is attributed to the different density of bioreceptors on the surface of PCs group G3 when 27 nM thrombin detection is performed in Section 3.1, as it was already discussed then.

**Figure 8:**
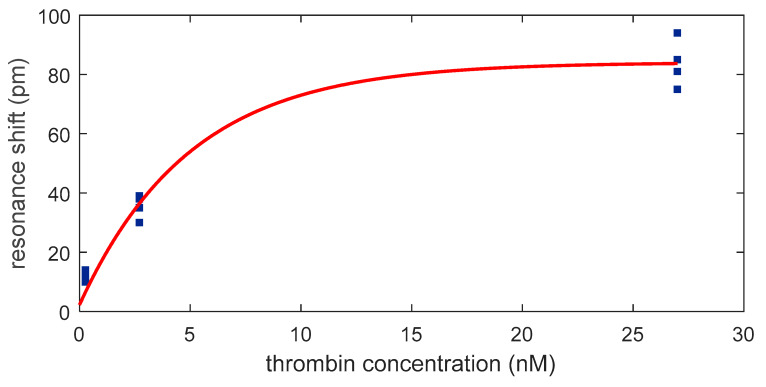
Dependence of the optical aptasensor response with the thrombin concentration.

By plotting the resonance shift against the concentration of thrombin, an exponential relation is obtained (Figure 8) with the correlation equation (R^2^=0.99):

(1) $$resonance\;shift\;\left(pm\right)=-81.8\;e^{-0.2002\;C_{thrombin}\left(nM\right)}+84.03$$

where $C_{thrombin}$ is the concentration of thrombin. This exponential curve shows a dynamic behavior from 270 pM to 27 nM and a linear behavior for concentrations below 2.7 nM. The correlation equation in the linear range (R^2^ = 0.99) is

(2) $$resonance\;shift\;\left(pm\right)=12.6\;C_{thrombin}\left(nM\right)+3.778$$

From this equation a sensitivity of 12.6 pm/nM is obtained. 

The LOD was calculated employing the equation [33]

(3) $$LOD=\bar{y_{bl}}+3\;\sigma_{bl}$$

where $\bar{y_{bl}}$ is the mean and $\sigma_{bl}$ is the standard deviation of the resonance shift experience by the PCs groups when BS is initially flowed. According to Equation (3), the estimated LOD of this aptasensor is 33.5 pM. Finally, the response time (defined here as the time that the sensor requires to experience half of the maximum resonance shift for a certain thrombin concentration) was calculated to be ~2 min.

## 4. Conclusions

In this work, the capability of a label-free optical aptasensor based on silicon 1D PC structures for the continuous monitor of increasing thrombin concentrations has been proved. Using this aptasensor, we have been able to detect thrombin concentrations ranging from 270 pM to 27 nM, thereby covering the nanomolar range corresponding to the initiation phase of thrombin generation that other thrombin generation assays cannot afford [3]. The sensitivity in the linear range is 12.6 pm/nM, the estimated LOD is 33.5 pM, and the response time is ~2 min. Other groups have published similar label-free assays with increasing concentrations, but the detected concentrations were higher [34,35,36], which indicates that our system has a better sensitivity, as far as our knowledge. On the other hand, other groups published the detection of similar thrombin concentrations, but employing dyes, long incubation times and without the possibility of monitoring the recognition of thrombin by TBA as it occurs [26,27]. 

## References

[1] Crawley J.T.B., Zanardelli S., Chion C.K.N.K., Lane D.A. (2007). The central role of thrombin in hemostasis. J. Thromb. Haemost..

[2] Mann K.G., Brummel K., Butenas S. (2003). What is all that thrombin for?. J. Thromb. Haemost..

[3] Wolberg A.S., Campbell R.A. (2008). Thrombin generation, fibrin clot formation and hemostasis. Transfus. Apher. Sci..

[4] Brummel K.E., Paradis S.G., Butenas S., Mann K.G. (2002). Thrombin functions during tissue factor-induced blood coagulation. Blood.

[5] Hockin M.F., Jones K.C., Everse S.J., Mann K.G. (2002). A model for the stoichiometric regulation of blood coagulation. J. Biol. Chem..

[6] Danforth C.M., Orfeo T., Everse S.J., Mann K.G., Brummel-Ziedins K.E. (2012). Defining the boundaries of normal thrombin generation: Investigations into hemostasis. PLoS ONE.

[7] ten Cate H., Hemker H.C. (2016). Thrombin Generation and Atherothrombosis: What Does the Evidence Indicate?. J. Am. Heart Assoc..

[8] Tripathy D., Sanchez A., Yin X., Luo J., Martinez J., Grammas P. (2013). Thrombin, a mediator of cerebrovascular inflammation in AD and hypoxia. Front. Aging Neurosci..

[9] Wojtukiewicz M.Z., Hempel D., Sierko E., Tucker S.C., Honn K.V. (2016). Thrombin—Unique coagulation system protein with multifaceted impacts on cancer and metastasis. Cancer Metastasis Rev..

[10] Remiker A.S., Palumbo J.S. (2018). Mechanisms coupling thrombin to metastasis and tumorigenesis. Thromb. Res..

[11] Duarte R.C.F., Ferreira C.N., Rios D.R.A., dos Reis H.J., das Carvalho M.G. (2017). Thrombin generation assays for global evaluation of the hemostatic system: Perspectives and limitations. Rev. Bras. Hematol. Hemoter..

[12] Kintigh J., Monagle P., Ignjatovic V. (2018). A review of commercially available thrombin generation assays. Res. Pract. Thromb. Haemost..

[13] Mohammadi Aria M., Erten A., Yalcin O. (2019). Technology Advancements in Blood Coagulation Measurements for Point-of-Care Diagnostic Testing. Front. Bioeng. Biotechnol..

[14] Deng B., Lin Y., Wang C., Li F., Wang Z., Zhang H., Li X.F., Le X.C. (2014). Aptamer binding assays for proteins: The thrombin example—A review. Anal. Chim. Acta.

[15] Adachi T., Nakamura Y. (2019). Aptamers: A review of their chemical properties and modifications for therapeutic application. Molecules.

[16] Zhang Y., Lai B.S., Juhas M. (2019). Recent advances in aptamer discovery and applications. Molecules.

[17] Hong P., Li W., Li J. (2012). Applications of aptasensors in clinical diagnostics. Sensors.

[18] Nguyen P.L., Sekhon S.S., Ahn J.Y., Ko J.H., Lee L., Cho S.J., Min J., Kim Y.H. (2017). Aptasensor for environmental monitoring. Toxicol. Environ. Health Sci..

[19] Pohanka M. (2019). Current trends in the biosensors for biological warfare agents assay. Materials.

[20] Karimi F., Dabbagh S. (2019). Gel green fluorescence ssDNA aptasensor based on carbon nanotubes for detection of anthrax protective antigen. Int. J. Biol. Macromol..

[21] Damborský P., Švitel J., Katrlík J. (2016). Optical biosensors. Essays Biochem..

[22] Garcia J., Sanchis P., Martinez A., Marti J. (2008). 1D periodic structures for slow-wave induced non-linearity enhancement. Opt. Express.

[23] Ruiz-Tórtola Á., Prats-Quílez F., González-Lucas D., Bañuls M.-J., Maquieira Á., Wheeler G., Dalmay T., Griol A., Hurtado J., García-Rupérez J. (2018). High sensitivity and label-free oligonucleotides detection using photonic bandgap sensing structures biofunctionalized with molecular beacon probes. Biomed. Opt. Express.

[24] Krauss I.R., Merlino A., Giancola C., Randazzo A., Mazzarella L., Sica F. (2011). Thrombin-aptamer recognition: A revealed ambiguity. Nucleic Acids Res..

[25] Ponce A.T., Hong K.L. (2019). A mini-review: Clinical development and potential of aptamers for thrombotic events treatment and monitoring. Biomedicines.

[26] Chen X., Li T., Tu X., Luo L. (2018). Label-free fluorescent aptasensor for thrombin detection based on exonuclease I assisted target recycling and SYBR Green I aided signal amplification. Sens. Actuators B Chem..

[27] Cho H., Baker B.R., Wachsmann-Hogiu S., Pagba C.V., Laurence T.A., Lane S.M., Lee L.P., Tok J.B.H. (2008). Aptamer-based SERRS sensor for thrombin detection. Nano Lett..

[28] Ruiz-Tórtola Á., Prats-Quílez F., González-Lucas D., Bañuls M.J., Maquieira Á., Wheeler G., Dalmay T., Griol A., Hurtado J., Bohlmann H. (2018). Experimental study of the evanescent-wave photonic sensors response in presence of molecular beacon conformational changes. J. Biophotonics.

[29] Oliverio M., Perotto S., Messina G.C., Lovato L., De Angelis F. (2017). Chemical Functionalization of Plasmonic Surface Biosensors: A Tutorial Review on Issues, Strategies, and Costs. ACS Appl. Mater. Interfaces.

[30] Schuck P., Zhao H. (2010). The role of mass transport limitation and surface heterogeneity in the biophysical characterization of macromolecular binding processes by SPR biosensing. Methods Mol. Biol..

[31] Manfrinato V.R., Zhang L., Su D., Duan H., Hobbs R.G., Stach E.A., Berggren K.K. (2013). Resolution limits of electron-beam lithography toward the atomic scale. Nano Lett..

[32] Petrova I., Konopsky V., Nabiev I., Sukhanova A. (2019). Label-Free Flow Multiplex Biosensing via Photonic Crystal Surface Mode Detection. Sci. Rep..

[33] Armbruster D.A., Pry T. (2008). Limit of blank, limit of detection and limit of quantitation. Clin. Biochem. Rev..

[34] Düzgün A., Maroto A., Mairal T., O’Sullivan C., Rius F.X. (2010). Solid-contact potentiometric aptasensor based on aptamer functionalized carbon nanotubes for the direct determination of proteins. Analyst.

[35] Bekmurzayeva A., Dukenbayev K., Shaimerdenova M., Bekniyazov I., Ayupova T., Sypabekova M., Molardi C., Tosi D. (2018). Etched fiber bragg grating biosensor functionalized with aptamers for detection of thrombin. Sensors.

[36] Coelho L., Marques Martins de Almeida J.M., Santos J.L., da Silva Jorge P.A., Martins M.C.L., Viegas D., Queirós R.B. (2016). Aptamer-based fiber sensor for thrombin detection. J. Biomed. Opt..

